# Crystal Structure of the RNA Recognition Motif of Yeast Translation Initiation Factor eIF3b Reveals Differences to Human eIF3b

**DOI:** 10.1371/journal.pone.0012784

**Published:** 2010-09-16

**Authors:** Sohail Khoshnevis, Piotr Neumann, Ralf Ficner

**Affiliations:** Department of Molecular Structural Biology, Institute of Microbiology and Genetics, Georg-August University Göttingen, Göttingen, Germany; Yale University, United States of America

## Abstract

**Background:**

The multi-subunit eukaryotic initiation factor3 (eIF3) plays a central role in the initiation step of protein synthesis in eukaryotes. One of its large subunits, eIF3b, serves as a scaffold within eIF3 as it interacts with several other subunits. It harbors an RNA Recognition Motif (RRM), which is shown to be a non-canonical RRM in human as it is not capable to interact with oligonucleotides, but rather interacts with eIF3j, a sub-stoichiometric subunit of eIF3.

**Principal Finding:**

We have analyzed the high-resolution crystal structure of the eIF3b RRM domain from yeast. It exhibits the same fold as its human ortholog, with similar charge distribution on the surface interacting with the eIF3j in human. Thermodynamic analysis of the interaction between yeast eIF3b-RRM and eIF3j revealed the same range of enthalpy change and dissociation constant as for the human proteins, providing another line of evidence for the same mode of interaction between eIF3b and eIF3j in both organisms. However, analysis of the surface charge distribution of the putative RNA-binding β-sheet suggested that in contrast to its human ortholog, it potentially could bind oligonucleotides. Three-dimensional positioning of the so called “RNP1” motif in this domain is similar to other canonical RRMs, suggesting that this domain might indeed be a canonical RRM, conferring oligonucleotide binding capability to eIF3 in yeast. Interaction studies with yeast total RNA extract confirmed the proposed RNA binding activity of yeast eIF3b-RRM.

**Conclusion:**

We showed that yeast eIF3b-RRM interacts with eIF3j in a manner similar to its human ortholog. However, it shows similarities in the oligonucleotide binding surface to canonical RRMs and interacts with yeast total RNA. The proposed RNA binding activity of eIF3b-RRM may help eIF3 to either bind to the ribosome or recruit the mRNA to the 43S pre-initiation complex.

## Introduction

Translation initiation in eukaryotes is intimately regulated by a set of proteins known as eukaryotic initiation factors (eIFs), which exploit diverse functions from positioning of the initiator tRNA in the ribosomal P-site to the recognition of mRNA [1]. The largest of these factors, eIF3, is a multi-subunit complex consisting of five stoichiometric subunits in yeast (eIF3a, b, c, g and i). Mammalian eIF3 has thirteen subunits, five of which are homologous to the yeast subunits of eIF3, hence believed to form the core of eIF3 and fulfill its essential functions [2]. The sixth subunit of eIF3 in yeast, eIF3j (Hcr1), is a loosely associated subunit and is suggested to play a role in recruitment of eIF3 to the ribosome [3]. In addition to its role in translation initiation, eIF3j is also involved in the biogenesis of ribosomal RNA [4]. It has also been suggested that eIF3j might serve a cross-talk function between translation initiation and termination by its interaction with translation termination factor eRF1 as well as eIF3 [5]. eIF3j interacts with the C-terminal domain (CTD) of eIF3a as well as the RNA recognition motif of eIF3b [6]. Solution structure of human eIF3b-RRM with a short peptide of eIF3j revealed the binding site for eIF3j to be located in a cleft between two helices of the RRM, distant from the exposed surface of the β-sheet which in other RRMs binds to the target RNA or DNA [7]. Interestingly, human eIF3b-RRM was shown to be a non-canonical RRM due to the high distribution of the negative charges around the canonical oligonucleotide binding site which makes it very unlikely to be able to accommodate any oligonucleotide [8]. Comparing the predicted isoelectric points of human and yeast eIF3b-RRM shows that in contrast to negatively charged human eIF3b-RRM, the yeast homolog is highly positively charged. Here, by using X-ray crystallography we studied the structure of yeast eIF3b-RRM and showed that it corresponds to the canonical RNA recognition motif. This domain also exhibited binding to the yeast total RNA, further confirming the proposed function. Nonetheless, determination of its specific physiological target remains out of the scope of this work. We further studied the thermodynamics of the interaction between eIF3b-RRM and eIF3j in yeast and showed that similar to human, this interaction is enthalpy driven. Given the similarity in surface charge distribution at the eIF3j binding site between yeast and human eIF3b-RRM as well as the conservation of the eIF3j peptide interacting with this domain, we propose that yeast applies the same mode of interaction with eIF3j as human.

## Results and Discussion

### Structure of Yeast eIF3b-RRM

**Figure 1:**
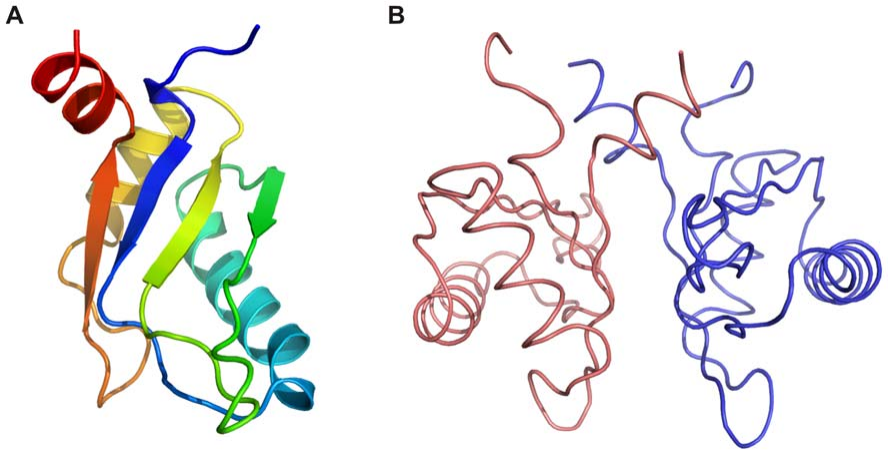
Overall structure of yeast eIF3b-RRM. (A) Overall fold of the yeast eIF3b-RRM showing the canonical β-α-β-β-α-β fold which at the C-terminus is followed by an extra helix which connects this domain to the rest of the protein (N- and C-termini are colored blue and red, respectively). (B) Relative orientation of two monomers in the asymmetric unit. Two monomers are held in place by interactions between residues in their β-sheets as well as the c-terminal helices. A 180° non-crystallographic symmetry axis exists between two monomers.

**Figure 2:**
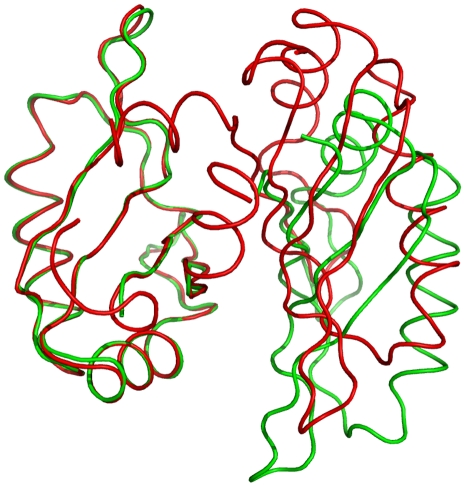
Different dimer formation between two different truncations of yeast eIF3b-RRM. Superimposition of the chains A of the yeast RRM^76–161^ (red) and RRM^76–170^ (green) indicates the difference in the relative orientation of the dimers between two molecules, indicating that the formation of the dimer is probably an artifact of the crystallization.

Two different truncations of the N-terminal domain of yeast eIF3b were designed based on homology to the published structure of its human ortholog [8]. Crystals of the shorter truncation (rsidues 76–161) diffracted to 2.6 Å. However, they were not easily reproducible as bunches of needles were obtained instead. The second construct encompassing residues 76–170 was designed to have an extra helix according to the secondary structure predictions. Highly reproducible crystals were obtained in space group P2_1_2_1_2_1_ with two molecules per asymmetric unit which diffracted to 1.25 Å. Crystallographic phases were obtained by molecular replacement using the structure of the sex-lethal RRM domain as search model. Data processing and refinement statistics are provided in Table 1. The structure is composed of four β-strands and two α-helices which are arranged in a β-α-β-β-α-β sequential order. The last β-strand is followed by a short α-helix which connects this domain to the rest of the protein (Figure 1A). This α-helix is absent in the NMR structure of human eIF3b-RRM. The two yeast eIF3b^76–170^ monomers occupying the asymmetric unit are related by a two-fold non-crystallographic symmetry axis, forming a closely packed dimer (Figure 1B). Analysis of the structure by PISA (http://www.ebi.ac.uk/msd-srv/prot_int/pistart.html) [9] shows that 14.8% (1805 Å^2^) of the total surface of the two molecules (12193 Å^2^) gets buried upon the dimer formation. Two monomers in the asymmetric unit are held in place by interactions between their β-sheets and between each β-sheet and the extended α-helix of the neighboring chain. Comparison of the relative orientation of the monomers between eIF3b-RRM^76–170^ and eIF3b-RRM^76–161^ showed that chain B in eIF3b-RRM^76–161^ has been displaced by ∼9 Å relative to the same chain in eIF3b-RRM^76–170^ (Figure 2). Therefore this dimer is most probably an artifact of the crystallization and not the functional unit of the protein in the solution. This is in agreement with the gel-filtration profile of the protein showing that it eluted from the column at a volume corresponding to the size of about 10 kDa (data not shown).

**Table 1:** Summary of the data collection and structure refinement.

	eIF3b-RRM^76–170^	eIF3b-RRM^76–161^
***A. Data collection***		
Space group	P2_1_2_1_2_1_	P2_1_2_1_2_1_
Unit cell parameter		
*a* (Å)	43.71	30.28
*b* (Å)	51.75	68.14
*c* (Å)	77.1	81.89
Resolution (Å)	30−1.25 (1.35−1.25)	34−2.6 (2.73−2.6)
Observed reflections	256057 (41259)	24819 (3276)
Unique reflections	48971 (8169)	5632 (771)
Completeness (%)	99.6 (99.4)	99.1 (94.6)
I/σI	12.42 (4.23)	10.8 (4.3)
R_merge_ a	8.1 (50.3)	11.1 (32.0)
Monomers in AU^b^	2	2
***B. Refinement***		
Resolution (Å)	1.2	2.6
R_work_ c	14.16	22.5
R_free_ d	16.75	27.25
Number of protein atoms in the AU	1538	1392
Number of solvent atoms	301	50
Number of ligand atoms and ions	20	0
Mean B-value(Å^2^)		
Protein	11.473	24.732
Solvent	23.931	16.929
Ligands and ions	30.611	
Rmsd from ideal		
Bond length (Å)	0.008	0.001
Bond angles (°)	1.247	0.480
Ramachandran plot(%)^e^		
Favoured	100	98.86
Outlier	0	0
Allowed	0	1.14
PDB codes	3NS6	3NS5

Values in brackets refer to the highest resolution shell.

a R_merge_  =  Σ_h_Σ_l_ | I_ih_-<I_h_> |/Σ_h_Σ_I_ <I_h_>, where <I_h_> is the mean of the observations I_ih_ of reflection h.

b AU stands for asymmetric unit.

c R_work_  =  Σ_hkl_| |F_obs_|–|F_calc_| |/Σ_hkl_ |F_obs_|, where F_obs_ and F_calc_ are the observed and calculated structure factors, respectively.

d R factor calculated for 5% randomly chosen reflections not included in the refinement.

e The geometry of the models was analyzed by Molprobity [26].

### Yeast eIF3b-RRM Interacts with eIF3j in a Similar Fashion to Its Human Homolog

**Figure 3:**
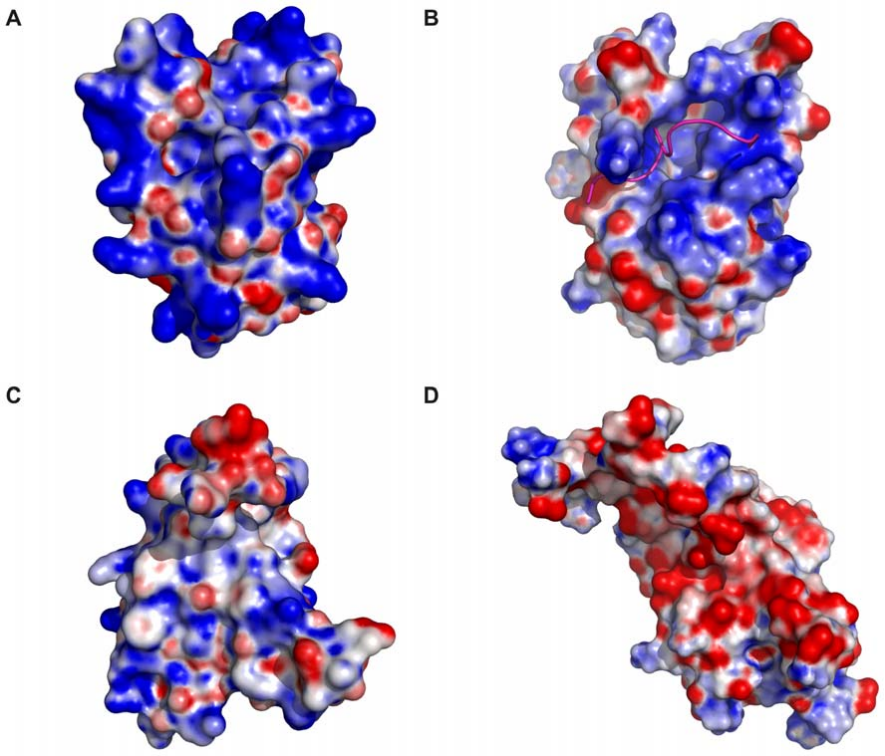
Surface charge distribution of yeast and human eIF3b-RRM. (A) Surface charge distribution of yeast eIF3b-RRM viewed from the α-helical side of the domain (distal to the oligonucleotide binding side) showing the accumulation of positive charges which would provide a suitable binding site for the negatively charged peptide of eIF3j. (B) Surface charge distribution of human eIF3b-RRM from the same view as (A). The short negatively charged peptide of eIF3j (magenta) sits in a basic cleft on eIF3b-RRM. (C). Surface charge distribution on the nucleotide binding side of the yeast eIF3b-RRM indicates the dominance of the positive over negative charges. This suggests that this motif can accommodate oligonucleotides. (D). Surface charge distribution on the nucleotide binding side of the humaneIF3b-RRM. Accumulation of acidic side-chains leaves no room for accommodation of any oligonucleotide.

**Figure 4:**
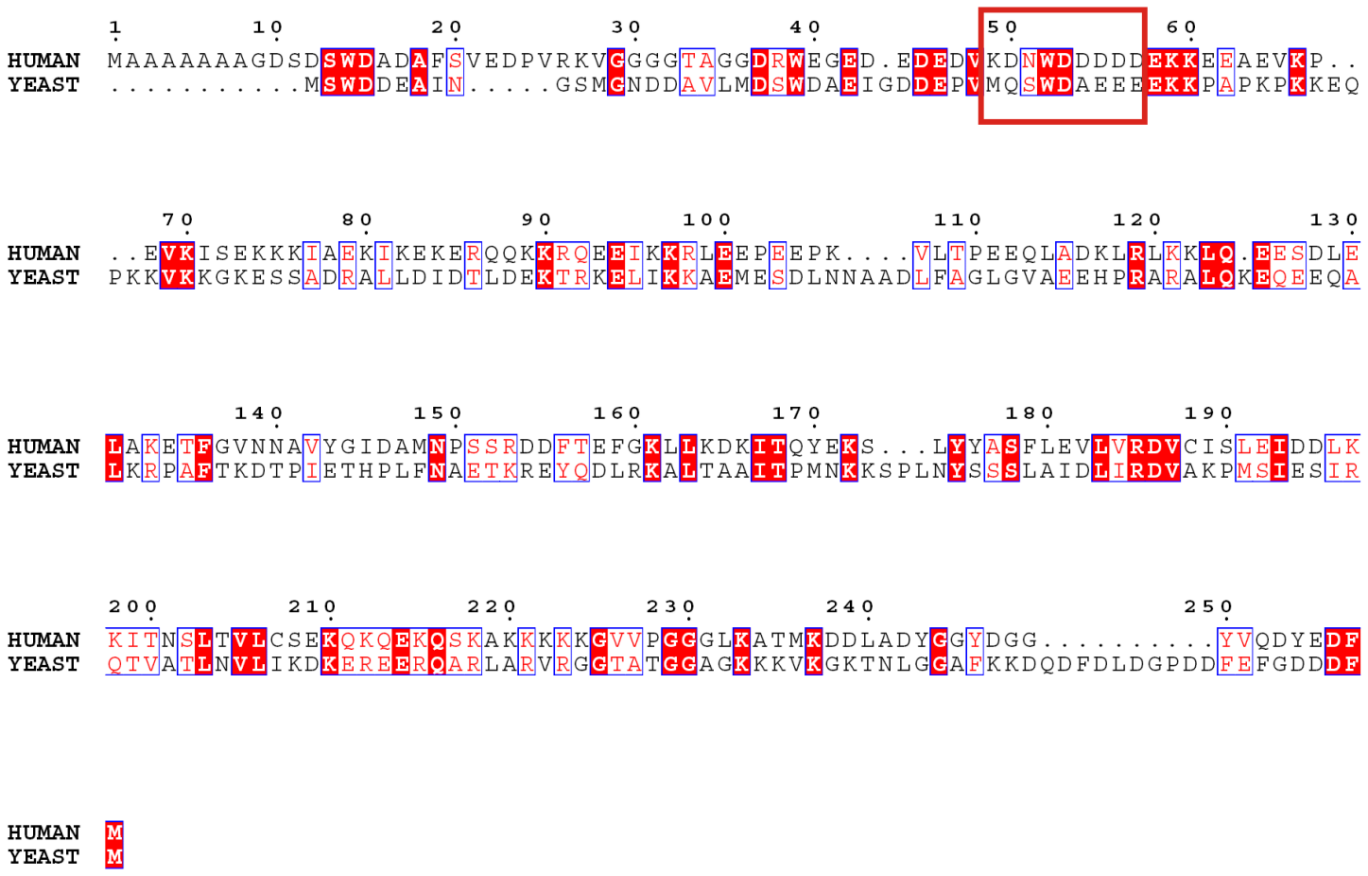
Sequence-based alignment of human and yeast eIF3j. The boxed sequence is the fragment which was solved in complex with human eIF3b-RRM (PDB code 2KRB). Conservation of the sequence of this region suggests the same mode of binding between yeast eIF3j and eIF3b-RRM. The alignment and the color representation were performed using ClustalW2 (www.ebi.ac.uk/Tools/clustalw2) [24] and ESPript 2.2 (www.espript.ibcp.fr/ESPript) [25] respectively.

**Figure 5:**
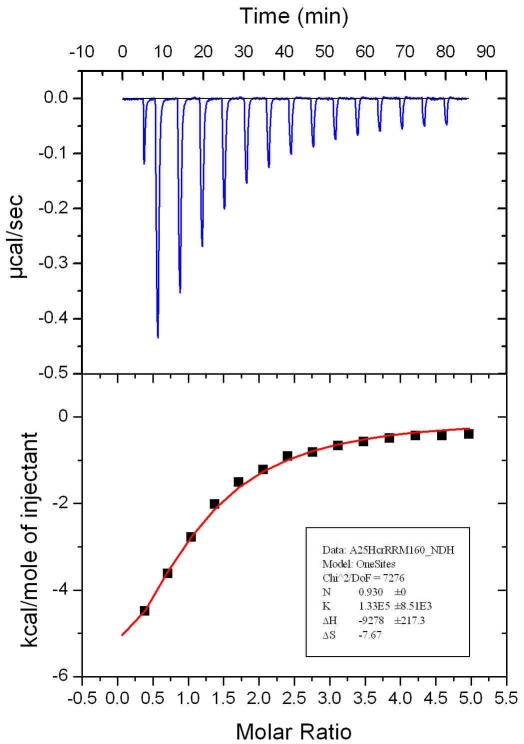
Isothermal calorimetric titration of eIF3j with eIF3b-RRM. The *upper panel* shows raw data of heat effect (in µcal·s^−1^) of 20-µl injections of 229 µM eIF3b-RRM into 1.5 ml of 10 µm eIF3j performed at 300 s intervals. The *lower panel* shows the fitted binding isotherms. The data points were obtained by integration of heat signals plotted against the molar ratio of eIF3b-RRM to eIF3j in the reaction cell. The *solid line* represents a calculated curve using the best fit parameters obtained by a nonlinear least squares fit.

Analysis of the surface charge distribution of the yeast eIF3b-RRM revealed a high degree of similarity to its human homolog with the surface made up of two α-helices packed against the β-sheet is rich in positively charged residues, creating an overall basic region distal to the accessible surface of the β-sheet (Figure 3A–B). The negatively-charged eIF3j peptide which interacts with human eIF3b-RRM [7] reveals amino acid conservation in yeast (Figure 4) which suggests the same mode of interaction between yeast eIF3b-RRM and eIF3j. Superposition of reported structure with that of human in complex with a short peptide of eIF3j (PDB code 2KRB) suggests that lysines 97, 105, 142, 147 and arginine 148 provide a positively charged surface for the accommodation of the negatively charged peptide of eIF3j. Another line of evidence for the same mechanism of the interaction between yeast 3b-RRM and 3j comes from the analysis of the thermodynamics of their interaction using isothermal titration calorimetry (ITC), showing that the *K_d_* of their interaction lies in the µM range, similar to the reported value for the human proteins. The raw ITC data and the integrated areas under each peak as a function of molar ratio of eIF3b-RRM to eIF3j are plotted in the upper and lower panels of Figure 5, respectively. The interaction is characterized by a large negative enthalpy change (Δ*H* = −9278±217.3 kcal.mol^−1^), indicating that similar to the human 3b-RRM, it is predominantly mediated through electrostatic interactions [8]. The binding curve is sigmoidal and best fitted to a single binding site model with ∼1∶1 stoichiometry, yielding a dissociation constant (*K_d_*) of 7.5±0.5 µM.

### Oligonucleotide Binding Potentials of Yeast eIF3b-RRM

**Figure 6:**
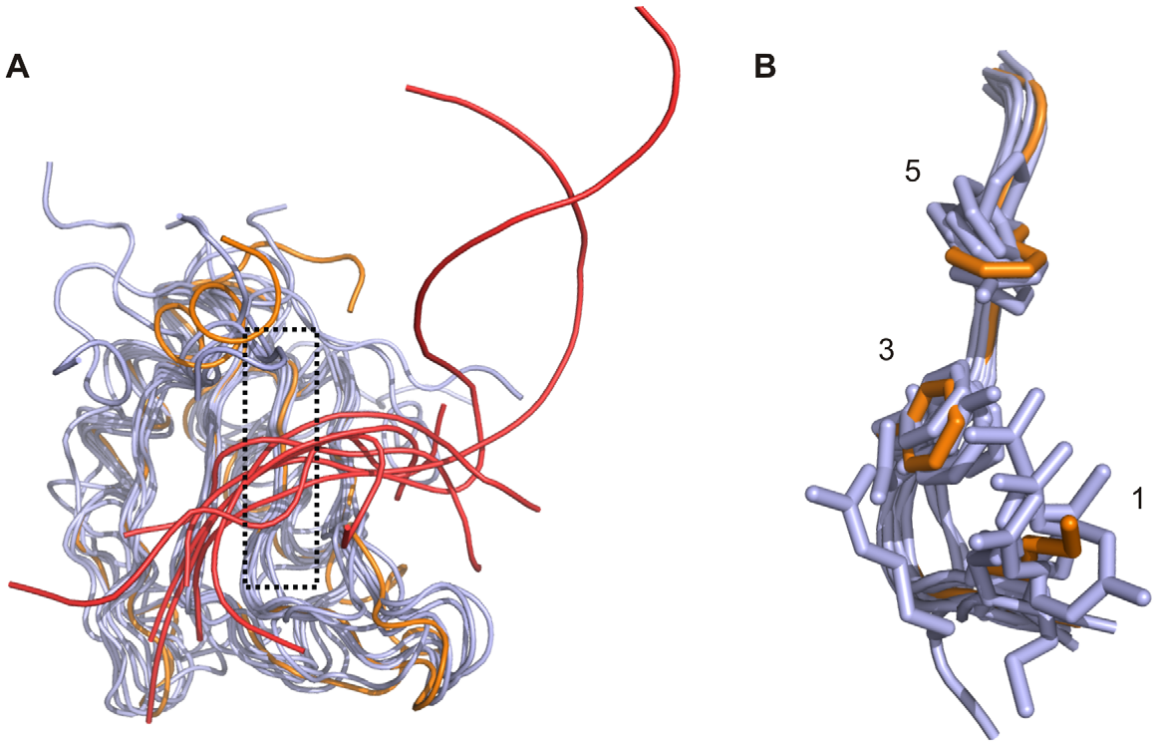
Superimposition of yeast eIF3b-RRM with several canonical RRMs bound to oligonucleotides from PDB. (A) Six different canonical RRMs (PDB codes 1B7F, 2AD9, 2KH9, 2RQC, 3D2W and 2UP1, all in grey) superimposed with yeast eIF3b-RRM (orange). As shown, oligonucleotides (red loops) occupy more or less the same position on the solvent exposed side of the β-sheet. (B) Three dimensional conservation of the elements of RNP1 (the black box on the panel A). The numbers correspond to the position of the amino acids in the motif [RK]_1_-G_2_-[FY]_3_-[GA]_4_-[FY]_5_-[ILV]_6_-X_7_-[FY]_8_.

**Figure 7:**
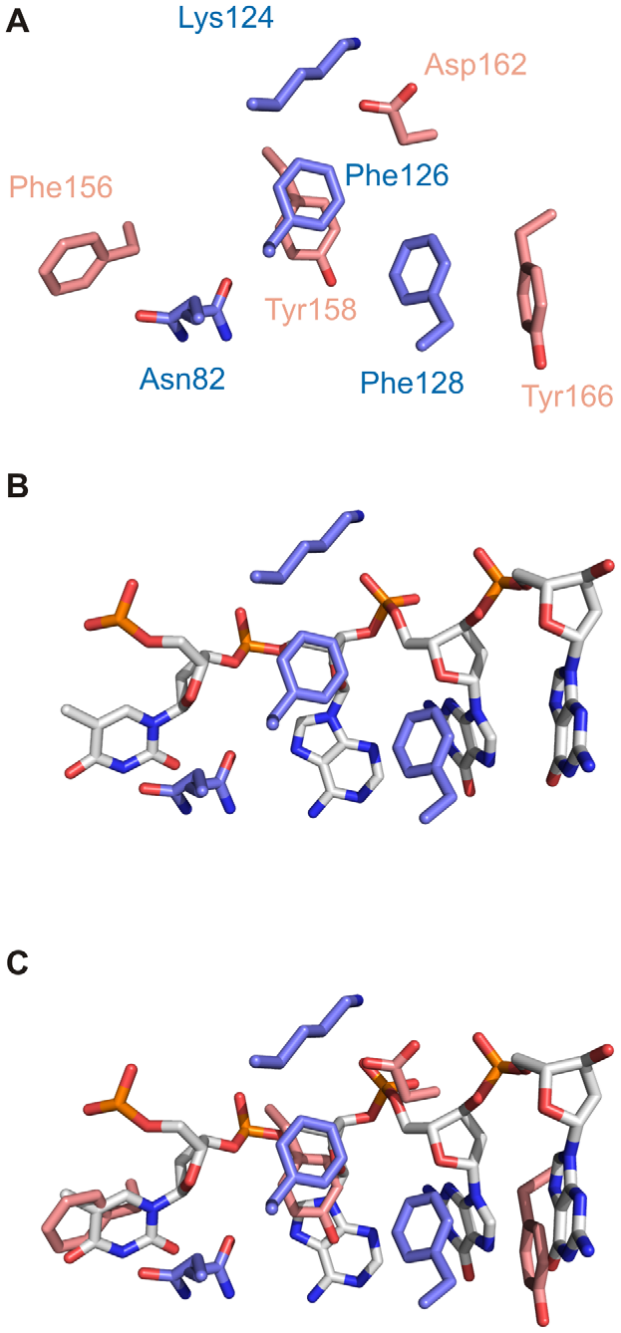
Critical interactions between two monomers in the asymmetric unit mimic the presence of an oligonucleotide. (A). The dimer of the RRM^76–170^ is held by several interactions, some of which involve components of the RNP1 (Lys 124, Phe 126 and Phe 128) or RNP2 (Asn 82). (B) Superposition of eIF3b-RRM with hnRNP A1 (UP1) in complex with single-stranded telomeric DNA (PDB code 2UP1) shows that different elements of RNP1 and RNP2 can interact with RNA bases (Asn82 in one of its alternative conformations and Phe128), sugar pocket (Phe126) or phosphate backbone (Lys124). (C) The interacting partners for the residues depicted in panel B assume the position of certain elements of the docked oligonucleotide; e.g. Asp 162 sits where the negatively charged phosphate group would sit, Phe 156 and Phe 166 occupy the position of bases of the oligonucleotide and Tyr 158 mimics the sugar pocket.

Analysis of the surface charge distribution of the yeast eIF3b-RRM revealed that in contrast to the human eIF3b-RRM, the solvent accessible surface of the β-sheet (distal to its surface which is packed against α-helices) is either neutral or positively charged (Figure 3C). This surface contains the “RNP1” motif, known for the canonical RRMs, comprised of the consensus sequence of [RK]-G-[FY]-[GA]-[FY]-[ILV]-X-[FY] [10]. Superposition of the structures of the monomer of yeast eIF3b-RRM with six canonical RRMs (PDB codes 1B7F, 2AD9, 2KH9, 2RQC, 3D2W and 2UP1) [11]–[16] bound to oligonucleotide (either RNA or DNA) suggests the conservation of these residues in three dimensions, which together with the appropriate charge distribution make should allow for binding to oligonucleotides (Figure 6). Surprisingly, the human homologue of this protein also shows three-dimensional conservation of this motif, however due to the accumulation of negatively charged residues on the surface of its β-sheet, it is not capable of accommodating an oligonucleotide (Figure 3D). Superpositioning of structures of yeast eIF3b-RRM with hnRNP A1 (UP1) complexed with single-stranded telomeric DNA (PDB code 2UP1) suggests it to bind in the same manner to an oligonucleotide, with Phe 126 stacking against the sugar pocket of one nucleotide and Phe 128 against the base of the next nucleotide, whilst the Lys 124 would neutralize the backbone phosphate (Figure 7B). Interestingly, comparison of the dimer of eIF3b-RRM with hnRNP A1 (UP1) shows that Tyr 158, Asp 162 and Phe166 of one monomer interact with the RNP1 motif of the second monomer in the crystal, mimicking the presence of the oligonucleotide (Figure 7A and C). Another motif known as “RNP2” with the sequence [ILV]-[FY]-[ILV]-X-N-L also plays a role in the interaction with the oligonucleotide, though it is not as conserved as the RNP1. For instance, in the structure of sex-lethal protein in complex with RNA (PDB code 1B7F) [11] the first RRM shows deviation from this consensus sequence, whereas the second RRM harbors the exact consensus motif. Therefore deviation of this motif from the consensus in yeast eIF3b-RRM does not exclude its oligonucleotide binding capacities. Nonetheless, in the superimposed structure, Asn82 which belongs to RNP2 motif in an alternative conformation is close to the putative position of the base of another nucleotide, which in the crystal is occupied by Phe156 from another monomer in the asymmetric unit (Figure 7C).

**Figure 8:**
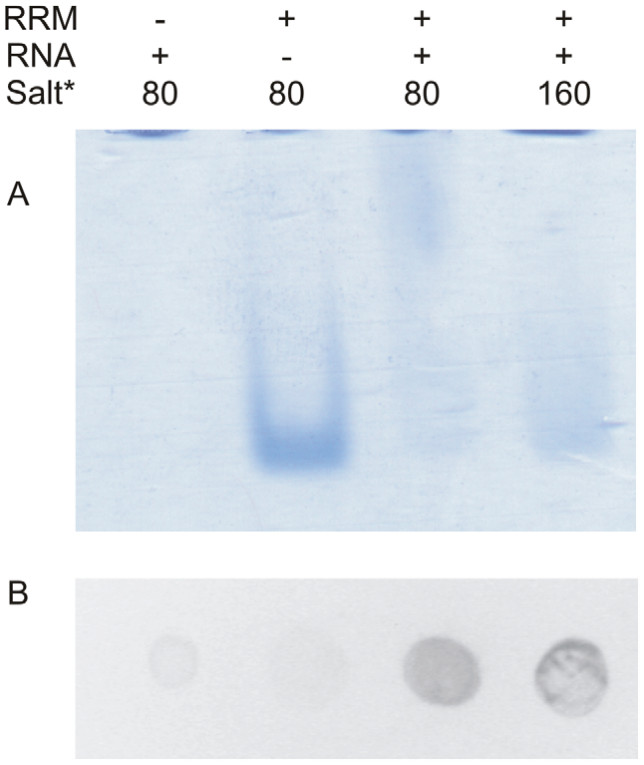
Binding studies between yeast IF3b-RRM and total RNA extract. (A). Electrophoretic mobility shift assay between RRM and total RNA indicates the upwards smearing of the RRM band upon interaction with RNA. The effect is more pronounced at lower salt concentration. (B). Filter binding assay confirms the results obtained by EMSA. Drops of filter binding assay are aligned under the corresponding lanes of EMSA. * stands for mM.

To test whether the proposed RNA binding activity occurs *in vitro*, the protein was mixed with the weight excess of the yeast total RNA to saturate the protein with RNA as much as possible and studied the interaction at two different salt concentrations by EMSA on a 5% native PAGE. Due to the overall positive charge of the protein, the gel had to be run in a positive-to-negative direction to get it into the gel. Results showed that in comparison to a sharp single band for the RRM alone, the addition of RNA resulted in smearing of the band upwards, indicating the formation of several RNA-protein complexes. The smearing was more pronounced at 80 mM than 160 mM salt (Figure 8A). To confirm these results, a filter binding assay was performed using the same complex preparations as for the EMSA but with the weight excess of the protein over the RNA to bind as much RNA as possible to the membrane for the sake of better detection. Results indicated higher binding of the yeast total RNA to the membrane in the presence of the RRM. Here, we also observed more binding at lower salt concentration (Figure 8B). Taken together, the results indicate the interaction between yeast eIF3b-RRM with its total RNA.

Although these results propose an RNA binding function of yeast eIF3b-RRM, determination of its exact RNA target is out of the scope of this work. However, it is tempting to speculate that the RNA binding ability conferred to eIF3 by this domain might serve to either recruit it to the ribosome via interactions with certain areas of the ribosomal RNA or facilitates its interaction with the mRNA, hence promoting the recruitment of the mRNA to the 43S pre-initiation complex. Although the human homolog of this domain has no RNA binding capability, this function may be fulfilled by additional components of human eIF3 which are missing in yeast.

## Materials and Methods

### Cloning

Yeast eIF3b-RRM spanning amino acid 76 to 161 or 170 and Hcr1were PCR amplified from yeast genomic DNA using following primers: 5′ CCGGGATCCGATCAGT ACATCGTCGTTAATG 3′ and 5′ CCGCTCGAGTTAGTCGTCAGAATTATATCTTT CAACA 3′ or 5′ CCGCTCGAGTTATTTCATAGTATAAAGAAACAAACGATGT 3′ for RRM^76–170^ and RRM^76–161^, respectively, and CCGGGATCCATGTCTTGGGACGA CGAAG and CCGCTCGAGTTACATAAAGTCGTCATCACCGAA for eIF3j. Resulting fragments were digested by BamH1 and Xho1 and ligated into the pGEX-6P-1 vector (GE Healthcare) which was digested with the same restriction enzymes. The ligation mixtures was then transformed into XL1-Blue cells (Stratagene) and plated on LB plates containing ampicillin. Positive transformants were determined by DNA sequencing and transformed into strain Rosetta2 (DE3) (Novagen).

### Protein expression and purification

For large-scale protein expression, 10 mL of preculture of each protein was prepared by resuspending several colonies of Rosetta2 (DE3) cells harboring either eIF3b-RRM or eIF3j in pGEX-6P-1 into LB medium supplemented with appropriate antibiotics at 37°C. Next day, 500 mL 2xYT media containing chloramphenicole and ampicilline was inoculated with 5 mL of each preculture and incubated at 37°C at 220 rpm. The protein expressions were induced with 0.2 mM IPTG at OD ∼0.5 and cells were subsequently transferred to 17°C. After 20 hours, cells were harvested and the resulted cell pellets were shock-frozen in liquid nitrogen. For protein purification, cells were resuspended in lysis buffer (300 mM NaCl, 50 mM Hepes pH 7.5, 5% Glycerol and 2 mM β-mercaptoethanol). Prior to the lysis, a protease mixture containing aprotinin, leupeptin and pepstatin was added to the suspension. Cell rupture was carried out by passing the cells six times through the microfluidizer (Microfluidics, Newton, US). Cell debris was removed by centrifugation at 30,000 g for 30 minutes on a JA-20 rotor (Beckman). The proteins were purified by applying the supernatants on a 5 mL GSTrap column (GE Healthcare) using Äkta prime (GE Healthcare). After loading the sample, the column was washed for two column volumes with wash buffer (2M LiCl, 50 mM Hepes pH 7.5, 5% Glycerol, 2 mM β-mercaptoethanol) to remove any bound oligonucleotide and then equilibrated back in lysis buffer. GST-fusion protein was eluted by washing the column with elution buffer (the same as lysis buffer plus 30 mM reduced glutathione). Fractions containing the fusion protein were pooled together and treated with PreScission protease (GE Healthcare) overnight to cleave off the GST. Fro RRM purification, this sample was concentrated the next day to 5 mL and loaded on a Superdex S-75 (26/60) column (GE Healthcare) pre-equilibrated in GF buffer (100 mM NaCl, 10 mM Hepes pH 7.5, 2 mM DTT). Fractions containing eIF3b-RRM were merged together and concentrated to 12 mg mL^−1^ and flash frozen in liquid nitrogen. For Hcr1 purification, the cleaved GST and the PreScission protease were removed by a second step of glutathione affinity chromatography and the GST-free Hcr1 sample was loaded on a Superdex S-75 (26/60) column pre-equilibrated in GF buffer. The eluant protein was concentrated to 17 mg mL^−1^ and flash frozen in liquid nitrogen.

### Crystallization, data collection and structure determination

Needle-shape crystals of eIF3b-RRM^76–170^ were obtained at 20°C using sitting drop method by mixing equal volumes of protein (11 mg.mL^−1^) with 30% PEG 4000, 200 mM Li_2_SO_4_ and 100 mM Hepes pH 8. Crystals of good diffraction quality grew after three days to the final size of 100*20*10 micrometer. Datasets were collected at the beamline ID23-2 at ESRF, Grenoble. The data set was processed in the space group P222 using XDS [17] and scaled to the final resolution of 1.25 Å. The phase problem was solved by molecular replacement using the crystal structure of *Drosophila melanogaster* sex-lethal protein (PDB code 3SXL) [18] as the search model in Phaser [19]. The initial model was further built and improved manually in Coot [20] and subsequently subjected to iterative steps of refinement in Phenix [21] and manual model building in Coot.

Plate-shape crystals of eIF3b-RRM^76–161^ were obtained by mixing the same volume of the protein (17 mg.mL^−1^) with the reservoir containing 33% PEG4000 and 0.1 M Na-citrate (pH 5.6) in sitting drop plates at 20°C. Good diffracting crystals appeared after two weeks. A dataset was collected on the home source beamline equipped with MAR345dtb detector mounted on a Micromax 007 generator operating with a copper target at 1.5417 Å. The dataset was indexed, scaled and reduced using XDS and SCALA [22] in the space group P 222 to the final resolution of 2.6 Å. The structure of eIF3b-RRM^76–170^ monomer was used as the search model in Phaser. This model was subsequently subjected to iterative steps of refinement in Phenix and manual model building in Coot.

Both structures are deposited at the Protein Data Bank with the accession codes 3NS5 (eIF3b-RRM^76–161^) and 3NS6 (eIF3b-RRM^76–170^).

### Isothermal titration calorimetry

Hcr1 and eIF3b-RRM^76–170^ were extensively dialyzed against ITC buffer (200 mM NaCl, 10 mM Hepes pH 7.5, 5% Glycerol) and concentrated to 10 and 229 µM, respectively. The experiment was performed on a VP-ITC calorimeter (Microcal). 20 µL aliquots of RRM were injected into the cell containing Hcr1 every 40 second, during which the titration peak returned to the base line. Separately, seven injections of the same concentration of RRM into the buffers were performed under the same conditions to determine the heat of dilution. The titration data were analyzed using the ORIGIN software to calculate the thermodynamics parameters.

### RNA binding assays

Yeast total RNA was extracted from strain InviScI (Invitrogen) using Nucleospin RNA II kit (Macherey-Nagel, Germany). 5% Acrylamide gel was made as described previously [23] To allow the detection by Coomassie Blue 2 µg of the protein was applied to 5 µg of the RNA in a reconstitution buffer [23] at the final volume of 10 µL. The salt concentration of the buffer was changed to obtain the final salt concentration of either 80 or 160 mM. As the control, two samples, one only with RRM and the other only with RNA were prepared in the final salt concentration of 80 mM. After 15 minutes of incubation at 25°C, samples were mixed with 2 µL of 6X loading dye (50% sucrose, 0.02% bromophenol blue, 0.02% xylene cyanol) and loaded on the native gel. The gel was run at 7–10 W in the cold room for 30 minutes in a positive-to-negative direction. Filter binding assay was performed by mixing 10 µg of the protein with 5 µg of the RNA in a final volume of 10 µL in the same buffers as for the native gel. After 15 minutes of incubation at 25°C, the whole sample was loaded as a drop on a nitrocellulose membrane connected to the vacuum. Each drop was washed two times with reconstitution buffer and stained with GelRed (BIOTIUM, USA).
